# Profiling of SARS-CoV-2 Subgenomic RNAs in Clinical Specimens

**DOI:** 10.1128/spectrum.00182-22

**Published:** 2022-03-21

**Authors:** Zigui Chen, Rita Way Yin Ng, Grace Lui, Lowell Ling, Chit Chow, Apple Chung Man Yeung, Siaw Shi Boon, Maggie Haitian Wang, Kate Ching Ching Chan, Renee Wan Yi Chan, David Shu Cheong Hui, Paul Kay Sheung Chan

**Affiliations:** a Department of Microbiology, Faculty of Medicine, The Chinese University of Hong Kong, Hong Kong Special Administrative Region, China; b Department of Medicine and Therapeutics, Faculty of Medicine, The Chinese University of Hong Kong, Hong Kong Special Administrative Region, China; c Department of Anaesthesia and Intensive Care, Faculty of Medicine, The Chinese University of Hong Kong, Hong Kong Special Administrative Region, China; d Department of Anatomical and Cellular Pathology, Faculty of Medicine, The Chinese University of Hong Kong, Hong Kong Special Administrative Region, China; e Jockey Club School of Public Health and Primary Care, Faculty of Medicine, The Chinese University of Hong Kong, Hong Kong Special Administrative Region, China; f Department of Paediatrics, Faculty of Medicine, The Chinese University of Hong Kong, Hong Kong Special Administrative Region, China; g Stanley Ho Centre for Emerging Infectious Diseases, Faculty of Medicine, The Chinese University of Hong Kong, Hong Kong Special Administrative Region, China; City University of Hong Kong

**Keywords:** RT-PCR, COVID-19, RNA-seq, SARS-CoV-2, subgenomic

## Abstract

SARS-CoV-2 transcribes a set of subgenomic RNAs (sgRNAs) essential for the translation of structural and accessory proteins to sustain its life cycle. We applied RNA-seq on 375 respiratory samples from individual COVID-19 patients and revealed that the majority of the sgRNAs were canonical transcripts with N being the most abundant (36.2%), followed by S (11.6%), open reading frame 7a (ORF7a; 10.3%), M (8.4%), ORF3a (7.9%), ORF8 (6.0%), E (4.6%), ORF6 (2.5%), and ORF7b (0.3%); but ORF10 was not detected. The profile of most sgRNAs, except N, showed an independent association with viral load, time of specimen collection after onset, age of the patient, and S-614D/G variant with ORF7b and then ORF6 being the most sensitive to changes in these characteristics. Monitoring of 124 serial samples from 10 patients using sgRNA-specific real-time RT-PCR revealed a potential of adopting sgRNA as a marker of viral activity. Respiratory samples harboring a full set of canonical sgRNAs were mainly collected early within 1 to 2 weeks from onset, and most of the stool samples (90%) were negative for sgRNAs despite testing positive by diagnostic PCR targeting genomic RNA. ORF7b was the first to become undetectable and again being the most sensitive surrogate marker for a full set of canonical sgRNAs in clinical samples. The potential of using sgRNA to monitor viral activity and progression of SARS-CoV-2 infection, and hence as one of the objective indicators to triage patients for isolation and treatment should be considered.

**IMPORTANCE** Attempts to use subgenomic RNAs (sgRNAs) of SARS-CoV-2 to identify active infection of COVID-19 have produced diverse results. In this work, we applied next-generation sequencing and RT-PCR to profile the full spectrum of SARS-CoV-2 sgRNAs in a large cohort of respiratory and stool samples collected throughout infection. Numerous known and novel discontinuous transcription events potentially encoding full-length, deleted and frameshift proteins were observed. In particular, the expression profile of canonical sgRNAs was associated with genomic RNA level and clinical characteristics. Our study found sgRNAs as potential biomarkers for monitoring infectivity and progression of SARS-CoV-2 infection, which provides an alternative target for the management and treatment of COVID-19 patients.

## INTRODUCTION

Severe acute respiratory syndrome coronavirus 2 (SARS-CoV-2), the virus that causes novel coronavirus disease 2019 (COVID-19), is an enveloped positive-sense single-stranded RNA virus belonging to the genus *Betacoronavirus*. SARS-CoV-2 together with severe acute respiratory syndrome coronavirus (SARS-CoV) and Middle East respiratory syndrome coronavirus (MERS-CoV) are classified under the family *Coronaviridae* (1, 2). SARS-CoV-2 carries 29,903 nucleotides, with the 5′-terminal two-thirds of the genome encoding two large nonstructural polyproteins (open reading frame 1a [ORF1a] and ORF1b) that are translated from the positive-strand genomic RNA and further cleaved into a total of 16 nonstructural proteins (NSPs) (3). Replication of the viral genome generates a complementary negative-sense genome length RNA that serves as a template for the synthesis of positive-strand genomic RNA (gRNA) and subgenomic RNAs (sgRNAs) (4). The sgRNAs contain a common 5′ leader sequence of approximately 70 nucleotides fused to different segments from the 3′ end of the viral genome that were likely generated from a paused negative-sense RNA synthesis occurring at the so-called transcription regulatory sequences (TRS) locating at the 3′ end of the leader sequence (TRS-L) as well as preceding each viral gene called the body (TRS-B), which results in discontinuous transcription of a nested set of positive-strand viral mRNAs for translation of four conserved structural proteins, namely, the spike (S), envelope (E), membrane (M) and nucleocapsid (N), and six accessory proteins (ORF3a, 6, 7a, 7b, 8 and 10) (5).

Coronavirus transcription is a discontinuous process, including a template switch during the synthesis of subgenomic negative-sense RNAs to add a copy of the leader sequence, which is regulated by multiple factors such as the extent of base-pairing between TRS-L/B, viral and cell protein-RNA binding, and high-order RNA-RNA interactions (4). Coronavirus RNA-dependent RNA synthesis is performed by a replication-transcription complex that is associated with the extensively rearranged intracellular membranes, including the so-called double-membrane vesicles (DMVs) and convoluted membranes (CMs), that somehow “protect” the synthesized RNAs from the stimulation of the host innate immune response. Most coronavirus subgenomic RNAs synthesized could be predicted *in silico* (6) and qualified *in vitro* (3). Using the Vero cell culture system, Kim et al., identified numerous discontinuous transcription events of SARS-CoV-2, with the N gene sgRNA being the most abundantly expressed transcript (5). Another study by Alexandersen et al. (7) used Ampliseq NGS to detect SARS-CoV-2 sgRNAs in 12 nasopharyngeal/oropharyngeal swabs revealed a different profile of sgRNAs from those observed by Kim et al. (5). Attempts to use sgRNA to identify active infection have produced diverse results (7–10). So far as we know, there is no report on profiling the full spectrum of SARS-CoV-2 transcriptome in a large cohort of clinical samples collected throughout infection.

In this work, we used the probe hybridization RNA-seq approach to delineate the full spectrum of SARS-CoV-2 sgRNAs in a large collection of respiratory samples and identified numerous known and novel discontinuous transcription events potentially encoding full-length, deleted, and frameshift proteins. We further designed real-time polymerase chain (RT-PCR) assays targeting 9 canonical sgRNAs to examine the dynamics of transcriptome on a series of consecutive respiratory specimens and stool samples.

## RESULTS

### Study subjects.

A total of 375 SARS-CoV-2 complete genomes, with a mean genome coverage of 99.6% ± 1.7% (85.4% to 100.0%) and a mean read depth of 20,779 ± 24,611× (32× to 167,012×), were obtained from the respiratory samples using probe hybridization RNA-seq (Data Set S1). Each respiratory sample was collected from one nonoverlapping individual patient. Based on the amino acid alignment of the S gene, 338 genomes (90%) contain a D614G mutation (an amino acid change from aspartic acid to glycine at position 614). No significant difference in viral load (median Ct value of 23.7; range: 12.2 to 32.5; *P* = 0.810), day of specimen collection from illness onset (median 6; range: 0 to 31 days; *P* = 0.740), illness severity (31 asymptomatic, 138 mild [no pneumonia], 133 moderate [pneumonia], 73 severe [oxygen supplementation]/critical [mechanical ventilation]; *P* = 0.414) and gender (203 female and 172 male; *P* = 0.220) was found between S-D614 and S-G614 genomes, while the former had younger average age than the latter (37 ± 19 versus 46 ± 22 years; *P* = 0.038) (Fig. S1). As expected, there was a negative association between viral load and day of collection from illness onset (*P ≤ *0.001). The disease progressed to critical was usually observed in patients with longer illness onset (8.3 ± 5.2 versus 5.6 ± 5.1 days; *P ≤ *0.001) or older subjects (64 ± 11 versus 39 ± 21 years; *P ≤ *0.001).

### Profile of canonical and noncanonical subgenomic RNAs.

**FIG 1:**
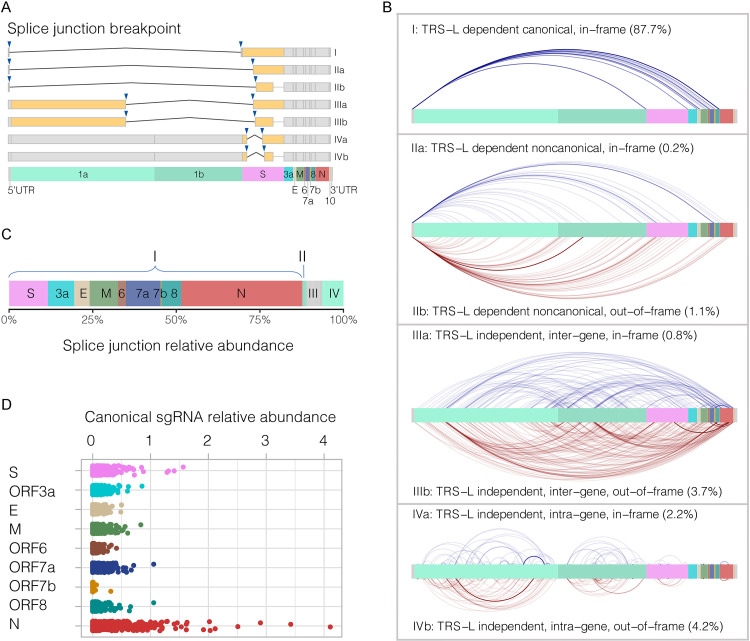
SARS-CoV-2 subgenomic RNAs in 375 respiratory samples obtained from individual COVID-19 patients using probe hybridization RNA-seq. (A) Model of discontinuous transcription events, including Type I of canonical subgenomic RNA mediated by TRS-L and TRS-B, Type II of noncanonical junction between leader TRS and a noncanonical 3′ terminus sites in the body of gene/ORF, Type III of the noncanonical junction with 5′- and 3′ terminus in the body of different genes/ORFs (intergene), and Type IV of noncanonical junction with 5′- and 3′ terminus in the body of a same gene/ORF (intragene). Subtypes “a” and “b” indicate reading frameshift relevant to the canonical gene. (B) Splice junction sites of discontinuous transcription events. The reads observed in 10 and more samples are shown, with curves in gradient colors showing differential relative abundance. (C) Transcript abundance spanning the junctions of the corresponding RNAs shown in panel B. (D) Expression level of SARS-CoV-2 canonical subgenomic RNAs inferred from the RNA-seq data. The relative abundance was normalized against the total viral RNA of each sample.

The depths of the 3′ terminus of the gRNA, such as ORF8, N, and ORF10, were substantially higher than that of the 5′ terminus (*P* ≤ 0.001) (Fig. S2), confirming the transcription of nested sgRNAs. Based on the breakpoints of the splice junction (Fig. 1A), all junction-spanning reads were divided into four patterns (Fig. 1B): Type I using the canonical template-switching mechanism mediated by transcription regulatory sequences in the leader (TRS-L) and the body (TRS-B) for discontinuous transcription to produce the major (canonical) sgRNAs (S, ORF3a, E, M, ORF6, ORF7a, ORF7b, ORF8, and N) (Table 1); Type II containing noncanonical fusion between TRS-L and an unexpected 3′ terminus site in the body of gene that encodes partial (IIa) or frameshifted proteins (IIb); Types III and IV showing noncanonical fusions with splice junction breakpoints located in the bodies of two different genes (intergene) and the body of the same gene (intragene), respectively. Normalization of junction-spanning reads against the total viral RNA of each sample showed that the canonical sgRNAs (Type I) represented most transcripts (87.7%) (Fig. 1C). Among them, the sgRNA N was most abundantly expressed (36.2%) followed by S (11.6%), ORF7a (10.3%), M (8.4%), ORF3a (7.9%), ORF8 (6.0%), E (4.6%), ORF6 (2.5%), and ORF7b (0.3%) (Fig. 1D). These canonical sgRNAs had a positively correlated expression to each other (Fig. S3). Of note, no splice junction sequence with expected structure and length of ORF10 was found in any surveyed sample.

**TABLE 1:** List of TRS-L dependent canonical subgenomic RNAs (sgRNAs) in SARS-CoV-2

sgRNA name	Splice junction		Junction length	Start codon offset	No. of detectable samples	Relative abundance	Open reading frame	
	5′	3′					5′	3′
S	66	21551	21486	11	323	50.1037	21563	25384
ORF3a	67	25381	25315	11	318	34.1264	25393	26220
E	70	26236	26167	8	298	19.9854	26245	26472
M	65	26467	26403	55	323	35.7022	26523	27191
ORF6	70	27040	26971	161	266	10.7121	27202	27387
ORF7a	67	27384	27318	9	325	44.6704	27394	27759
ORF7b	72	27761	27690	−6	163	0.9584	27756	27887
ORF8	66	27883	27818	10	307	25.7614	27894	28259
N	65	28254	28190	19	358	155.4361	28274	29533

**FIG 2:**
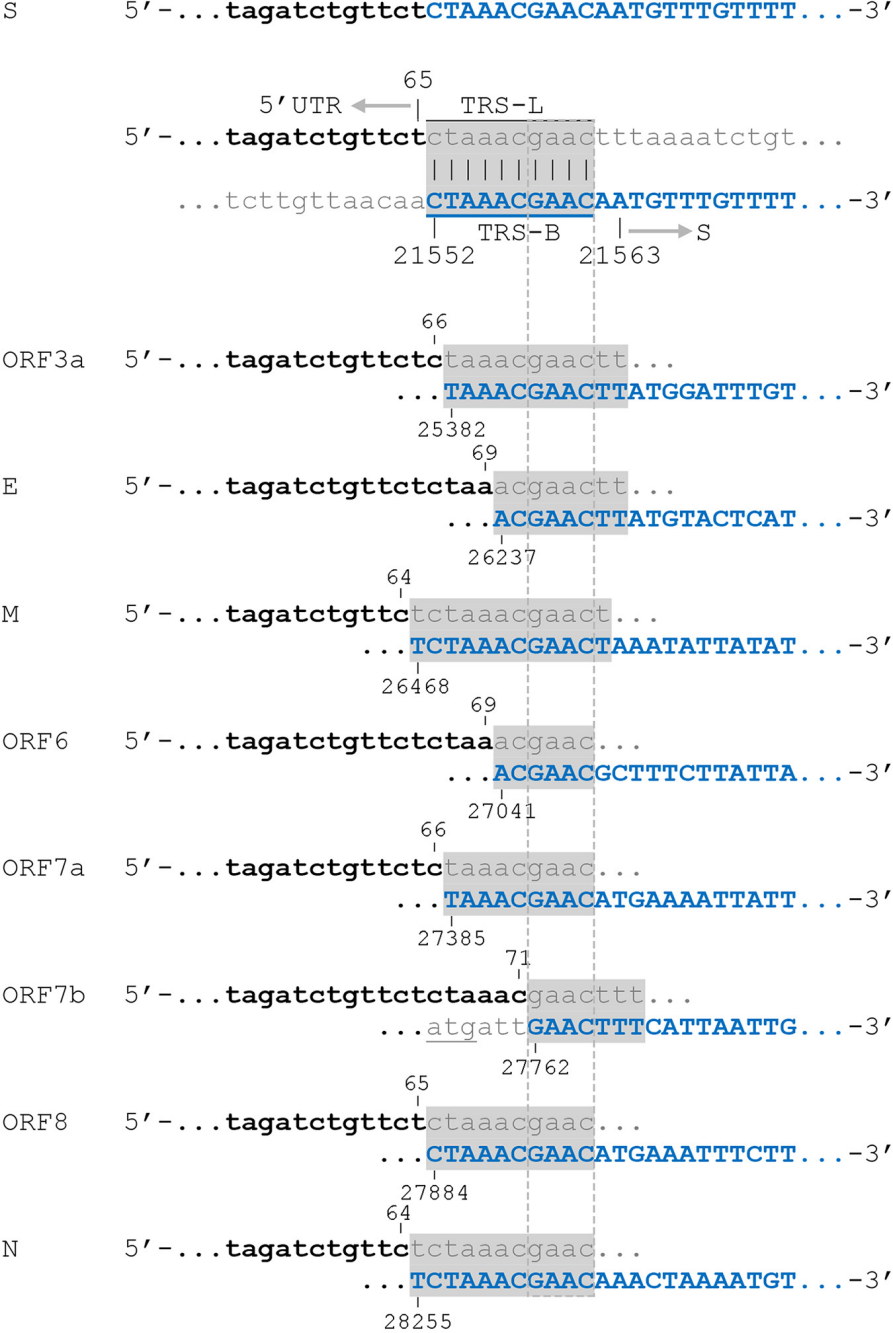
Consensus sequence (CS) alignment between transcription-regulator sequences in the leader (TRS-L) and the descent to each gene/ORF (TRS-B).

The canonical sgRNAs contained short consensus nucleotide sequences (CS) between TRS-L and TRS-B, ranging between 6 and 12 bp, whereas a 4-bp sequence (GAAC) was universally observed, suggesting a potential of complementarity-guided template switching of junction sites in all known canonical sgRNAs (see the box in Fig. 2). The stability of the extended duplex between the TRS-L and the complement of the TRS-B is critical for the synthesis of sgRNAs. In line with this, we found that subgenomic genes containing more extended base-matching sequences between TRS-L/B, such as N and M rather than ORF6 or ORF7b, exhibited a more efficient transcription. Besides the predominant forms, alternative splice junctions expressing canonical sgRNAs were observed in a small proportion of samples with much lower relative abundances. For example, the junction-spanning reads of 69 | 28262 for the sgRNA N were found in 164 samples with the cumulated relative abundance of 0.94 compared to the main form of 65 | 28254 (358 samples, cumulated relative abundance of 155.44) (Data Set S2). However, some of these transcripts might be subjected to sequencing errors, which warrants further validation.

Noncanonical transcripts (Types II, III, and IV) had a much lower abundance (12.3%) compared to canonical sgRNAs (87.7%). These frameshifted or deleted subgenomic RNAs may translate novel proteins with shorter or fused forms with functions and significance to be further explored. Of note, we detected 8 noncanonical sgRNAs that expressed at a higher level than the annotated full-length ORF7b (Fig. S4).

### Profile of canonical sgRNAs in respiratory samples of 375 individual patients.

**FIG 3:**
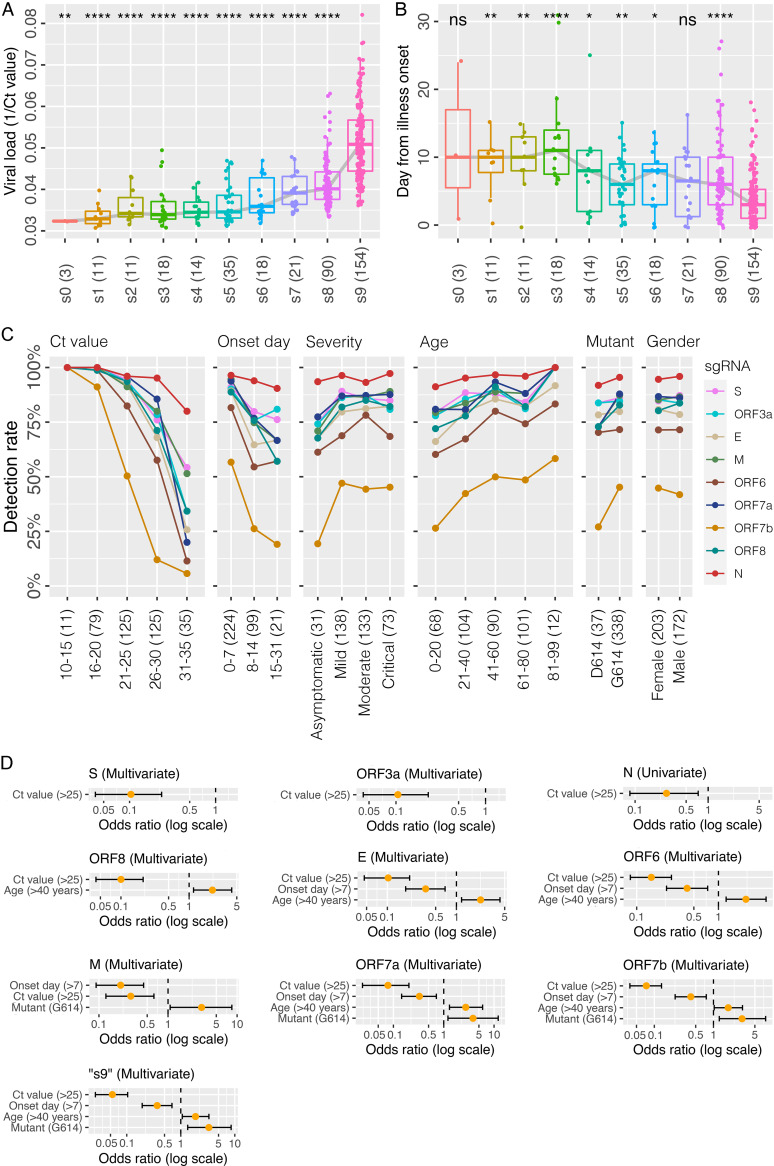
Differential expression of canonical subgenomic RNAs in respiratory samples inferred from RNA-seq data. (A) Detection of canonical subgenomic RNA patterns positively associated with viral load measured by the genomic RNA N. “s0” to “s9” indicates the gene number of detectable canonical subgenomic RNAs. For example, “s9” indicates a full set of 9 canonical subgenomic RNAs, while “s0” means no detectable canonical subgenomic RNA. The number of detectable samples of each subgenomic RNA pattern was listed in the bracket. Mann-Whitney U test was performed between “s9” and partial subgenomic RNA pattern, with *P* values indicating *, *P* ≤ 0.05; **, *P* ≤ 0.01; ***, *P* ≤ 0.001; ****, *P* ≤ 0.0001; ns, no significance. (B) Association between the detected canonical subgenomic RNA patterns and the day of collection from illness onset. (C) The detection rate of individual subgenomic RNA related to different patient characteristics. (D) Univariate and multivariate regression analyses of patient characteristics in detecting individual canonical subgenomic RNAs and the full set pattern (see details in Fig. S7).

We then compared the differential expression profiles of canonical sgRNAs revealed by RNA-seq. Overall, 154 out of 375 samples (41%) harbored splice junction sequences (read number ≥2) of a full set of 9 known canonical sgRNAs (designed as pattern “s9” in Fig. 3A), which showed a significant positive association with the viral load (median Ct value: 19.9; range: 12.2 to 27.7) compared to samples containing 8 or less canonical sgRNAs (“s1” to “s8”) (median Ct value: 26.3; range: 15.9 to 32.5; *P ≤ *0.001). Three samples, with Ct values between 30.9 and 31.0, did not harbor any detectable canonical sgRNA (“s0”). Furthermore, samples harboring a full set of 9 canonical sgRNAs (“s9”) were usually collected earlier compared to samples harboring a partial set of canonical sgRNAs (median days of 4.2 versus 7.5; *P ≤ *0.001) (Fig. 3B).

Consistent with the transcript expression levels (Fig. 1D), the sgRNA N was the most frequently detected and was found in 95% of samples (358/375), followed by ORF7a (86%), S (86%), M (86%), ORF3a (85%), ORF8 (82%), E (79%) and ORF6 (71%); while ORF7b being the least (43%, 163/375). In line with this, the majority (93%, 84/90) of “s8” samples lacked sgRNA ORF7b, and 57% (12/21) of “s7” was missing both sgRNA ORF7b and ORF6 (Fig. S5).

The changes in detection rates among the 9 canonical sgRNAs in association with viral load, day of collection from illness onset, disease severity, age, and viral mutant are shown in Fig. 3C and S6. As the level of the gRNA decreased (from low to high Ct values of genomic N RT-PCR, 10 to 15 → 16 to 20 → 21 to 25 → 26 to 30 → 31 to 35), the detection rate of sgRNA ORF7b dropped dramatically (100% → 91% → 50% → 12% → 6%). A similar response was exhibited by ORF6. In contrast, the sgRNA N was much less sensitive to viral load changes and maintained a high detection rate (100% → 100% → 96% → 95% → 80%) throughout a wide range of viral load. A sharp drop (≥25%) in detection rates of sgRNA ORF7b, ORF6, and E was also observed in samples collected late (≥8 days), younger subjects (≤20 years), those with milder disease, and probably S-D614 prototype. In Fig. 3C, a significantly lower level of sgRNA7b was associated with the asymptomatic, but not mild/moderate/critical disease (19% versus >44%).

The univariate and multivariate odds ratios for the detection of each subgenomic RNA with respect to viral load (indicated by Ct value of genomic N RT-PCR), days of sample collection from symptom onset, the disease progressed to pneumonia (moderate/critical/severe), S-614D/G variant and gender are shown in Fig. S7. The detection of most sgRNAs, except N, showed an independent association with viral load (Fig. 3D). Furthermore, an independent association of detection with age, sample collection from the onset, and S-614D/G mutant were also observed for the full set of 9 canonical sgRNAs (“s9”). Such independent association was reproduced by sgRNA ORFs 7a and 7b (Fig. 3D).

### Profile of canonical sgRNAs in serial respiratory and stool samples of 10 patients revealed by RT-PCR.

To examine the changes in canonical sgRNA profile during infection, we collected 124 serial upper and lower respiratory and stool samples from 10 patients (designated Patient A to J) during their hospitalization. All samples were tested positive by a diagnostic RT-PCR targeting genomic RNA for N (gRNA N). These samples were collected between 2 and 47 (median day of 11) days after illness onset, including 45 upper respiratory, 37 lower respiratory, and 42 stool specimens. They were examined by 9 assays of RT-PCR with each targeting a specific sgRNA encoding S, ORF3a, E, M, ORF6, ORF7a, ORF7b, ORF8, and N, respectively (Table 2). No RT-PCR assay for potential sgRNA ORF10 was designed in this work.

**TABLE 2:** Primers and probes used in real-time PCRs for the detection of 9 canonical subgenomic RNAs (sgRNAs)

Subgenome	Type	Position^*a*^	Sequence (5′–3′)	Primer length	%GC	Amplicon length
All sgRNAs	DNA probe	49–73	FAM-TCTTGTAGATCTGTTCTCTAAACGA-BHQ1	25	36.0	-
All sgRNAs	Forward Primer	27–46	ACAAACCAACCAACTTTCGA	20	40.0	-
S	Reverse Primer	21642–21623	GCAGGGGGTAATTGAGTTCT	20	50.0	130
ORF3a	Reverse Primer	25457–25437	TCCTTGATTTCACCTTGCTTC	21	42.9	116
E	Reverse Primer	26326–26307	GCAAGAATACCACGAAAGCA	20	45.0	133
M	Reverse Primer	26544–26525	TAGTACCGTTGGAATCTGCC	20	50.0	115
ORF6	Reverse Primer	27113–27094	ACTGTATGCAGCAAAACCTG	20	45.0	116
ORF7a	Reverse Primer	27444–27425	AAGCTCACAAGTAGCGAGTG	20	50.0	100
ORF7b	Reverse Primer	27818–27798	AACAAGGAATAGCAGAAAGGC	21	42.9	102
ORF8	Reverse Primer	27949–27929	TCTTGGTGAAATGCAGCTACA	21	42.9	105
N	Reverse Primer	28341–28322	GAATCTGAGGGTCCACCAAA	20	50.0	125

a Position refers to the prototype sequence (NC_045512).

**FIG 4:**
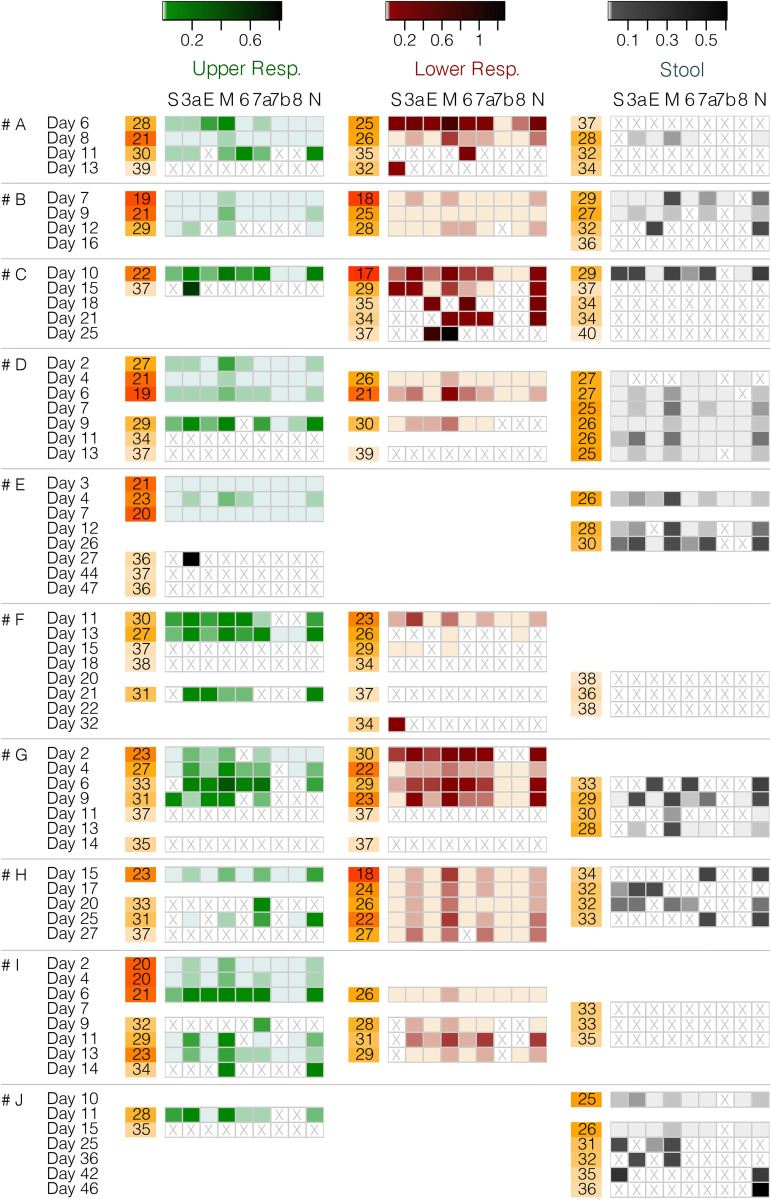
Detection of SARS-CoV-2 canonical subgenomic RNAs on serial samples (45 upper respiratory, 37 lower respiratory, and 42 stool specimens) from 10 COVID-19 patients using RT-PCR. Heatmap shows the relative concentration of each canonical subgenomic RNA against the genomic RNA N (2^ΔCt^). “X” indicates a negative result. The number at the beginning of each sample shows the Ct value of RT-PCR targeting the genomic RNA N.

A few patterns of the sgRNA profile were observed (Fig. 4). The first pattern was demonstrated by Patients A, B, and C, where the diagnostic PCR targeting genomic N revealed positive results from upper and lower respiratory and stool samples throughout the first 2 to 3 weeks after symptom onset. However, a full set of 9 sgRNAs (“s9”) was only detected in upper and lower respiratory samples for up to 10 days, and none in the stool samples. The second pattern was exhibited by Patient D who had a full set of sgRNAs (“s9”) detected from all three sites, which might indicate shedding of active viruses from both respiratory and gastrointestinal tracts. Patients E and F appeared to have a long duration of positivity based on diagnostic PCR targeting genomic RNA. However, a full set of sgRNAs were only detected up to day 7 and day 11 of Patient E and F, respectively. Patients G and H represented a pattern where a full set of sgRNAs were mainly detected from the lower respiratory tract which could be a source of genomic RNA fragments shed in the upper respiratory and gastrointestinal tracts. Patient I had the full set sgRNA pattern reappeared in the upper respiratory tract. Whereas, Patient J appeared to have prolonged fecal positivity revealed by diagnostic genomic PCR up to 46 days after illness onset, none of the 6 serial stool samples, including the earliest one taken on day 10 had a full set of sgRNAs.

**FIG 5:**
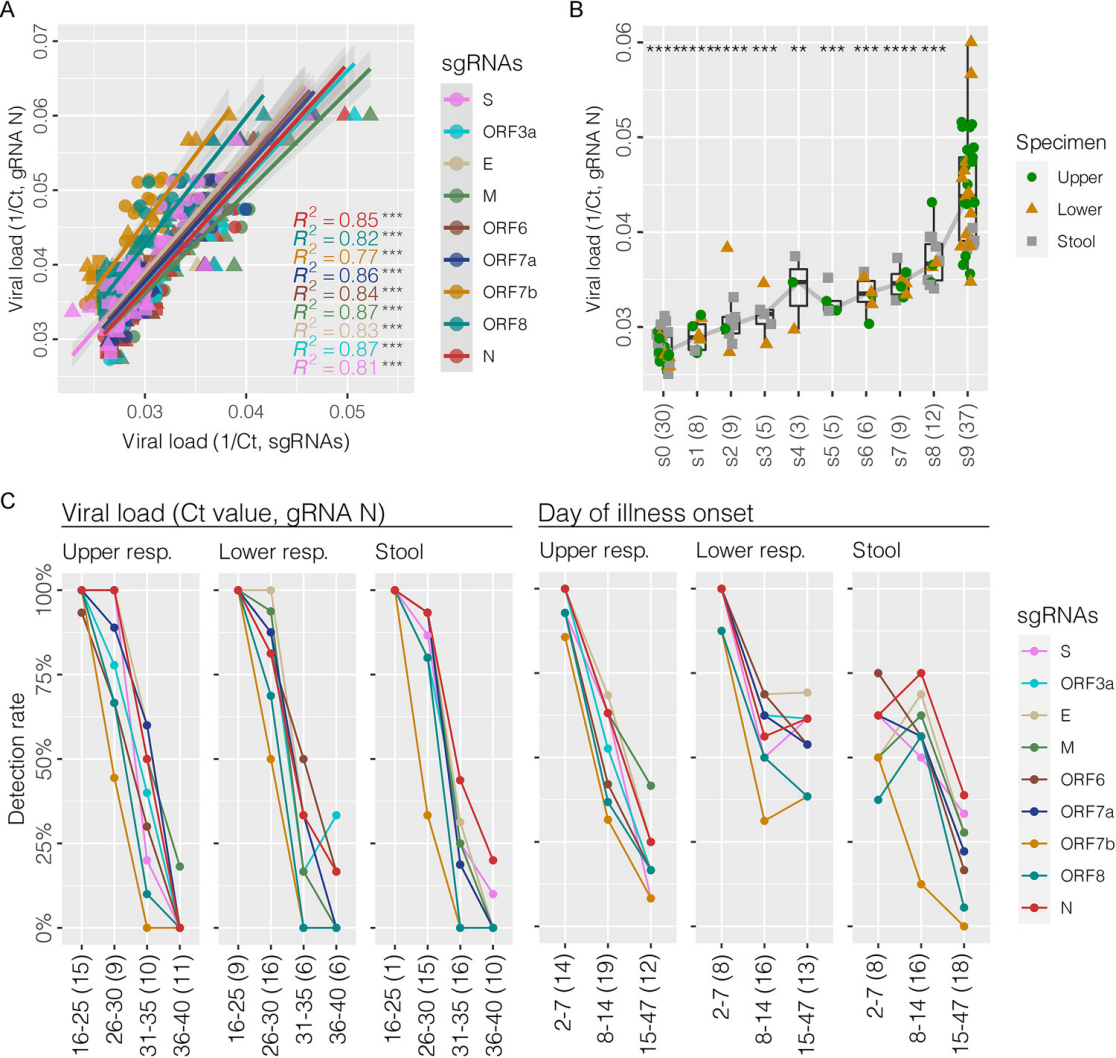
Profile of canonical subgenomic RNAs using RT-PCR in association with viral load and day of specimen collection from illness onset. (A) Scatterplot of correlation analysis showing a positive association between genomic and subgenomic RNAs. (B) Detection of canonical subgenomic RNA patterns positively associated with viral load measured by the N genomic RNA. “s9” indicates a full spectrum of 9 canonical subgenomic RNAs, while “s0” means no detectable canonical subgenomic RNA. The number of detectable samples of each subgenomic RNA pattern was listed in the bracket. Mann-Whitney U test was performed between “s9” and partial subgenomic RNA pattern, with *P* values indicating *, *P* ≤ 0.05; **, *P* ≤ 0.01; ***, *P* ≤ 0.001; ****, *P* ≤ 0.0001; ns, no significance. (C) Detection rate of each subgenomic RNA by RT-PCR assay in different specimen types in association with viral load indicated by genomic RNA N Ct values, and the timing of specimen collection in days after illness onset.

The concentration, as inferred from 1/Ct, of genomic and subgenomic RNAs detected from serial clinical specimens showed a high degree of correlation (*R^2^* ranged from 0.77 to 0.87; *P ≤ *0.001) (Fig. 5A). When different specimen types were compared, however, the sgRNAs in stool samples exhibited a much lower degree of correlation with the levels of genomic RNA (Fig. S8A). We were able to detect a full set of 9 canonical sgRNAs (“s9”) in 43% (16/37) of lower respiratory samples followed by upper respiratory samples (37.8%, 17/45), and the lowest in stool samples (10%, 4/42) (Fig. 5B and Fig. S8B). On the other hand, the proportion of samples without any detectable sgRNA (“s0”) was highest for stool samples (33.3%, 14/42), followed by upper respiratory samples (24.4%, 11/45) and lower respiratory samples (13.5%, 5/37). Consistent with the finding from RNA-seq data, the samples with detectable full set “s9” by RT-PCR had higher levels of genomic RNA (median Ct: 23; range:17 to 29) compared to partial-positive ones (median Ct: 33; range: 23 to 40; *P ≤ *0.001) (Fig. 5B), although the association was less strong for stool samples (Fig. S8B). The chance of detecting a full spectrum of sgRNAs from stool samples collected within 10 days of collection from illness onset was significantly lower than those of upper and lower respiratory samples (positive rate for stool: 20%, 3/15; upper respiratory: 70%, 14/20; and lower respiratory: 78.6%, 11/14; *P ≤ *0.01) (Fig. S8C).

During the switching from full set positive to partial positive for sgRNA profile, the sgRNA ORF7b was the most sensitive marker being the first or one of the first to become negative in 24/28 (85.7%) sets of serial samples (Fig. S9). Similarly, when the gRNA level decreased or when the collection time from onset increased, the sgRNA ORF7b became the first transcript to drop to a low detection rate (Fig. 5C).

## DISCUSSION

Previous studies reported the presence of a 5′ terminus leader 72-nt sequence in SARS-CoV-2 that mediates the leader-to-body splice fusion by switching the transcription-regulator sequences in the leader (TRS-L) to the “body” sequence (TRS-B) of the downstream genome for discontinuous transcription (4, 5). In this work, we used the probe hybridization RNA-seq approach to delineate the complete profile of SARS-CoV-2 subgenomic RNAs in respiratory samples collected from 375 individual COVID-19 patients. Besides known canonical subgenomic RNAs, numerous discontinuous transcription events producing partial, frameshifted, and fused RNAs potentially translating novel proteins were detected, revealing a highly complex landscape of SARS-CoV-2 RNA synthesis. These novel proteins, if being confirmed for the presence by long-read sequencing, may be studied further for potential features in viral life, transmissibility, and host immune evasion. Whether some of these sgRNAs are unique to SARS-CoV-2 or conserved in other pathogenic respiratory syndrome coronaviruses also warrants further examination.

In line with previous reports in cell culture and respiratory samples (5, 7), our RNA-seq data set and RT-PCR showed a highly variable expression of the 9 known canonical subgenomic RNAs. ORF10 probably does not express since no subgenomic reads were found for its corresponding transcript. Although one study using the ribosome footprint densities indicated the presence of a few translation initiation signals in the ORF10 region (3), further work is needed to delineate how ORF10 are translated and what functional role this protein has. Our data also support the importance of the complementarity between TRS-L and TRS-B in regulating the expression of subgenomic RNAs. A 4-nucleotide consensus sequence (GAAC) was universally observed in all observed canonical subgenomic RNAs and was concatenated by extra overlapping sequences with variable length. The subgenomic RNAs containing extended base-pairing sequence may facilitate stability of template switching via physical proximity between the TRS-L and a TRS-B during discontinuous transcription to promote efficient RNA-dependent RNA polymerase (RdRp) transfer (11). Interestingly, we observed a higher level of sgRNAs for N, S, M, ORF7a, and ORF8a for the S-D614G variant suggesting a more efficient expression or more active replication. SARS-CoV-2 S protein mediates virus attachment to the host cell surface receptors to promote virus-cell fusion and entry (12). The M protein defines the shape of the viral envelope and was reported to attenuate host antiviral immunity and enhance viral replication (13). The N protein is a multivalent RNA-binding protein critical for viral replication and genome packaging (14). The coronavirus N protein is also required for efficient subgenomic RNA transcription (15). Although the detailed mechanism and the interaction between SARS-CoV-2 subgenomic RNAs need further investigation, emerging data indicate that the S-D614G variant can promote virion spike density and infectivity (16), mediate membrane fusion (17), increase virus susceptibility to neutralization (18), produce higher infectious titers (19), suggesting a potential link between efficient RNA transcription and enhanced pathogenicity in SARS-CoV-2 S-D614G variants.

Whether sgRNAs can serve as a marker of viral activity in SARS-CoV-2 infection remains controversial (7–9). Our observations suggest that there is such potential. Samples exhibiting a full set of sgRNAs were mainly collected early in the course of illness, and an independent association between the detection of sgRNAs and clinical characteristics was observed, which supports the importance of early initiation of antiviral treatment rather than by the time the patient deteriorate and become critical. We also found that respiratory samples had a higher transcription expression in prevalence and abundance compared to stool samples. While this observation may suggest that SARS-CoV-2 shed to the gastrointestinal tract are probably rendered noninfectious, a direct measurement of infectious viral titer from stool samples is needed to verify this interpretation as the lower levels of sgRNAs in stool samples might be due to the inherent nature of stool samples or other unknown reasons. It is a major limitation that data on infectious viral titer were not able in this study. Further studies combing genomic-and subgenomic-specific RT-PCR assays, cell culture, and even animal models are needed to advance our understanding and provide an evidence-based clinical application of subgenomic RNA measurement.

Overall, the sgRNA N showed the highest abundance and prevalence, but the least clinical value in terms of correlation with clinical characteristics. In contrast, sgRNA ORF7b had the lowest abundance and prevalence but was the most sensitive marker being the first to become negative among serial samples. Furthermore, sgRNA ORF7b was found to be the most appropriate single sgRNA marker to indicate the presence of a full set of canonical sgRNAs in clinical specimens. The sgRNA ORF7b also reproduced the associations with viral load, age of the patient, and viral variant as observed for the full set sgRNA (“s9”). These findings support that real-time RT-PCR assay(s) targeting one or a few sensitive subgenomic RNAs (e.g., sgRNA ORF7b, ORF6, and/or E) can be a feasible means to provide an objective marker to stratify the need for isolation.

In conclusion, our study used RNA-seq and RT-PCR to delineate the expression profile of SARS-CoV-2 sgRNAs in many respiratory and stool samples. The expression profile of canonical sgRNAs was associated with genomic RNA level and clinical characteristics. In particular, sgRNA ORF7b was found to be the most sensitive surrogate marker to indicate the presence of a full set of canonical sgRNAs. sgRNA has the potential to be an objective biomarker for monitoring infectivity and progression of SARS-CoV-2 infection, thus providing a guide to triage patients for isolation and treatment.

## MATERIALS AND METHODS

### Ethics approval and patient recruitment.

In the first part, we analyzed 375 respiratory samples collected from individual patients using probe hybridization RNA-seq. In the second part, we designed RT-PCR assays targeting the 9 canonical sgRNAs on a total of 124 serial upper and lower respiratory swabs and stool samples collected from 10 patients. All patients provided written consent, and the study was approved by The Joint Chinese University of Hong Kong – New Territories East Cluster Clinical Research Ethics Committee.

### SARS-CoV-2 complete genome sequencing.

Total RNA was extracted from clinical samples using the QIAamp Viral RNA Minikit (Qiagen, Germany), and tested for SARS-CoV-2 RNA by RT-PCR targeting the genomic version of gene N as described before (20). Respiratory samples with Ct values lower than 32 were selected for SARS-CoV-2 whole-genome sequencing. In brief, the RNA was treated with DNase I, and subsequently, the human rRNA and globin genes were removed using QIAseq FastSelect rRNA and globin mRNA removal kit (Qiagen, Germany). The resulting RNA was converted to double-stranded cDNA by KAPA HyperPrep kit (Roche, USA), and the library was then prepared using Swift 2S Turbo DNA Library kit (Swift Biosciences, USA). To increase the proportion of viral reads relative to host genome contaminants, the libraries were further hybridized with SARS-CoV-2 capture probes provided by IDT (Integrated DNA Technologies, USA) and sequenced on Illumina NexSeq 500 (Illumina, USA) at the Core Utilities of Cancer Genomics and Pathobiology (CUCGP) at Department of Anatomical and Cellular Pathology of the Chinese University of Hong Kong, using 150 bp paired-end reads.

### Bioinformatics data analysis.

Illumina raw reads were proceeded for quality control to remove adapters and low-quality sequences using Trimmomatic v0.39 (21), and further filtered for human genome contaminants (GRCh38) using HISAT2 v2.2.0 (22). A reference-based assembly (accession number NC_045512) was performed to profile SARS-CoV-2 transcriptome using STAR v2.7.9a (23), with specific parameters as described before (5). Consensus sequences were called using bcftools v1.9 (24) and manually checked for ambiguous sites. The SARS-CoV-2 genomes with ≥80% completeness and ≥30× total RNA mean coverage were retained for splice junction annotation.

### Splice junction annotation.

The splice junction (SJ) reads were categorized by the positions of 5′- (the first position) and 3′ terminus (the end position) of deletion. An SJ was labeled as a leader-to-body junction (also called TBS-L dependent canonical SJ) when the 5′ terminus of the deletion was mapped to a genomic position between 55 and 85 nt, and the 3′ terminus of the deletion was located to the upstream of the first AUG of the gene/ORF. If 5′ terminus was in the 5’UTR region while 3′ terminus was within the body of gene/ORF, the SJ was marked as TBS-L dependent noncanonical junction. The TBS-L independent SJ was labeled as intrajunction and interjunctions when 5′- and 3′ terminus positions were located within the body of the same or different gene/ORF, respectively. In the case where the 5′ terminus was in the 5’UTR region, the frame matching of the noncanonical sgRNA was identified by the first appearance of the 3′ terminus in the spliced gene/ORF. When the 5′ terminus was in a known gene/ORF, we checked whether the concatenated sequence generates a protein product with the same reading frame as a canonical gene/ORF of the spliced 3′ terminus.

### RT-PCR for canonical subgenomic RNAs.

Nine primer-probe sets were designed to quantify SARS-CoV-2 canonical sgRNAs (S, ORF3a, E, M, ORF6, ORF7a, ORF7b, ORF8, and N) (Table 2). These assays share the same forward primer and probe, combined with a unique subgenomic-specific reverse primer. The RT-PCRs contained 5 μL of TaqMan Fast Virus 1-Step Master Mix (Thermo Fisher, Foster City, CA) in a final reaction volume of 25 μL. The primer and probe concentrations used were 0.2 μM for ORF3a, E, M, ORF6, ORF7a, and N; and 0.4 μM for S, ORF7b, and ORF8.

### Statistical analysis.

Comparison of viral loads and read abundance between groups were performed using Mann-Whitney U test, Kruskal-Wallis rank-sum test, or linear regression analysis as appropriate, and with two-tailed *P ≤ *0.05 considered statistically significant. Regression correlation analysis was applied to evaluate the association between viral load and days of collection from illness onset. Sequencing read coverage, subgenomic profiling, and splice junctions were visualized using in-house developed scripts in R v3.6.2 and ggplot2 v3.3.5.

### Availability of data and materials.

All short reads assembled to the reference genome have been deposited to the National Center for Biotechnology Information (NCBI) Sequence Read Archive (SRA) (Bioproject ID: PRJNA778445).
