# Supplementary material for: Profiling of SARS-CoV-2 Subgenomic RNAs in Clinical Specimens
Source: Microbiol Spectr. 2022 Mar 21;10(2):e00182-22. doi: 10.1128/spectrum.00182-22 (PMC9045320; doi:10.1128/spectrum.00182-22)
Supplement: SUPPLEMENTAL FILE 1 — Supplemental material. Download SPECTRUM00182-22_Supp_1_seq8.pdf, PDF file, 6.9 MB [file spectrum00182-22_supp_1_seq8.pdf]

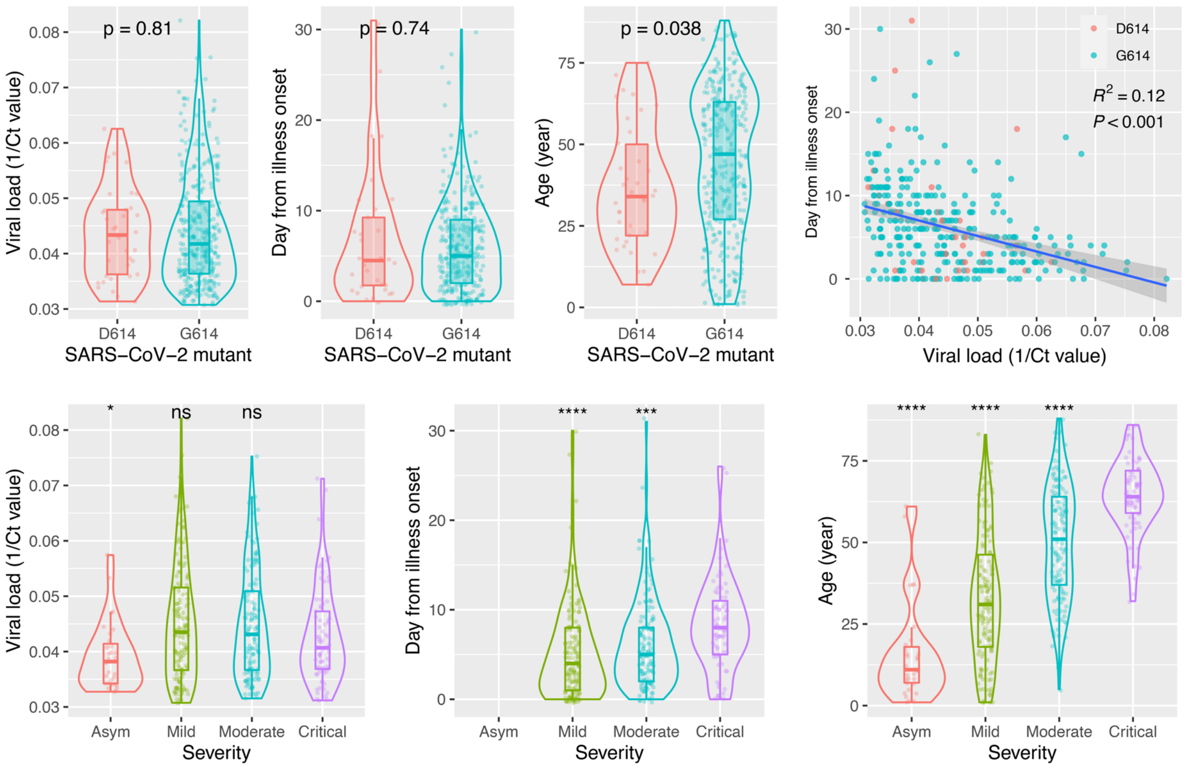

Figure S1. Comparison of viral load, day of collection from illness onset and age between patients with different viral mutant and severity outcome. Mann-Whitney U test was performed between critical patients and other severity groups, with p values indicating * p ⩽ 0.05, ** p ⩽ 0.01, *** p ⩽ 0.001, **** p ⩽ 0.0001, ns no significance. Asym, asymptomatic; Mild, mild without pneumonia; Moderate, moderate with pneumonia; Critical, severe (oxygen supplementation) /critical (mechanical ventilation).

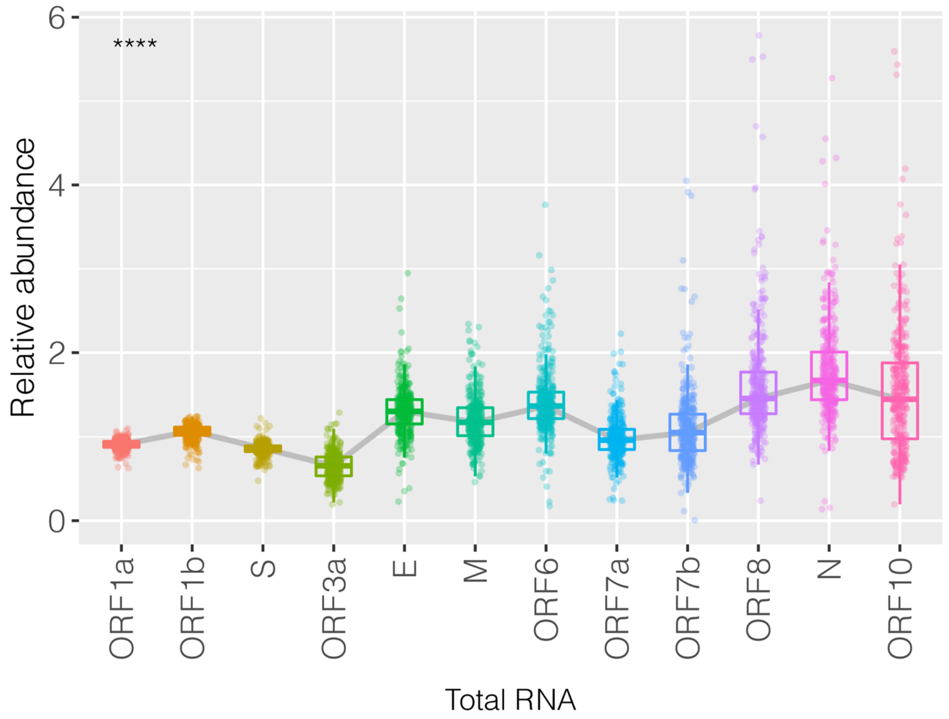

Figure S2. Relative abundance of total RNA encoding each gene against the average abundance of the whole genome.

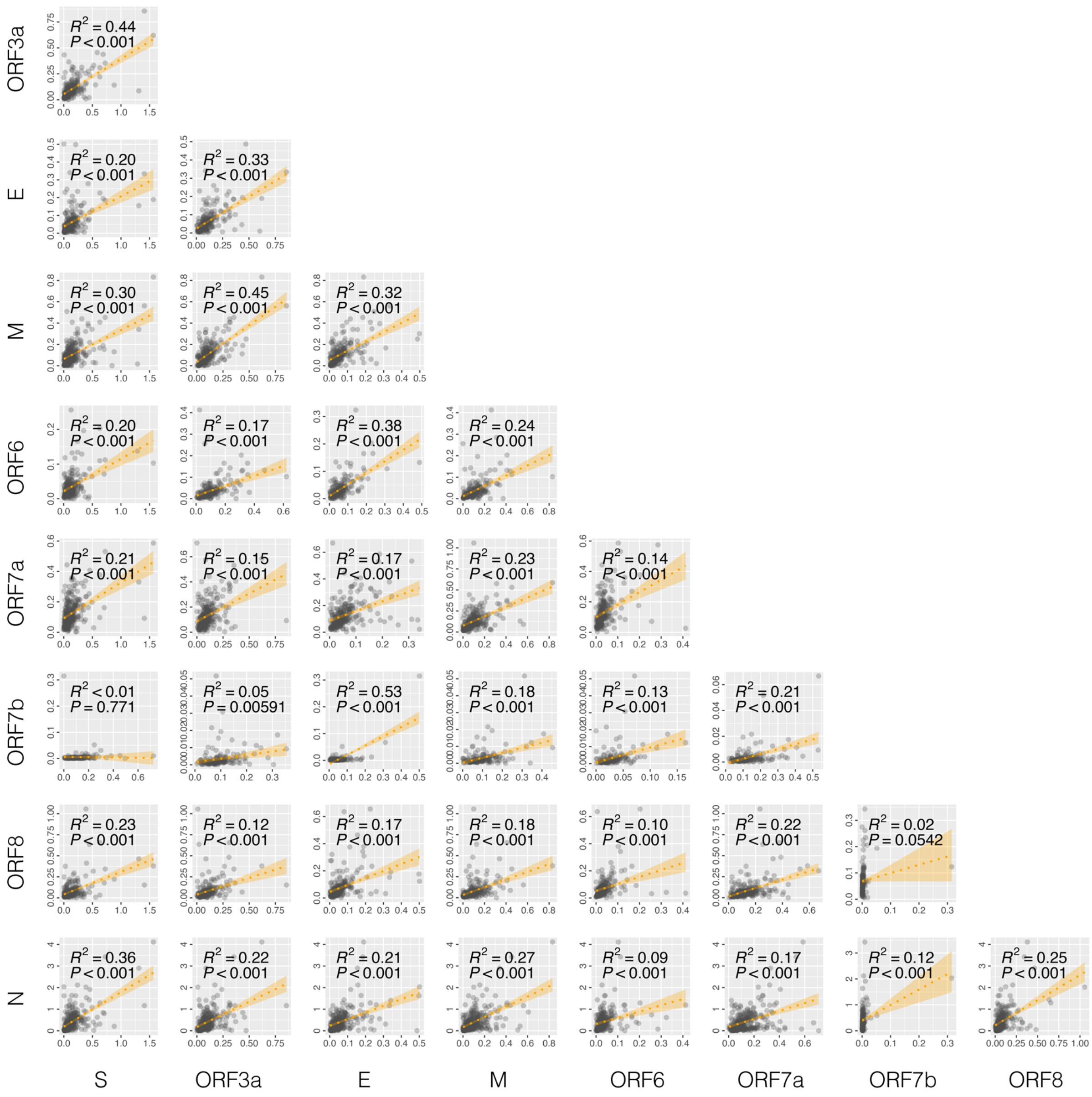

Figure S3. Pairwise correlation of relative abundance of canonical subgenomic RNAs inferred from RNA-seq data.

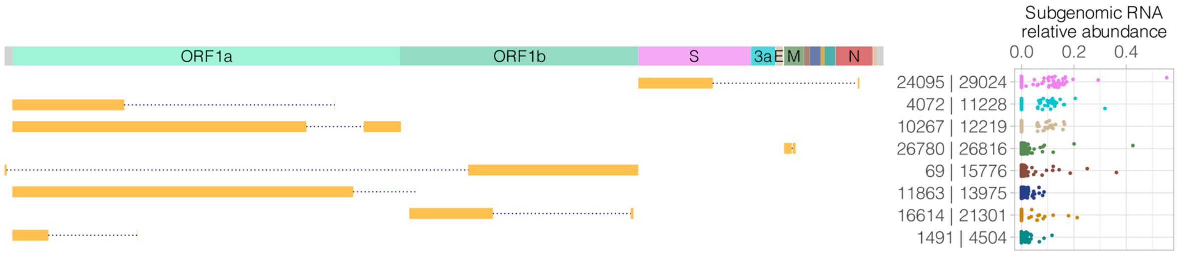

Figure S4. Relative abundance of noncanonical subgenomic RNAs that expressed at a higher level than the annotated full-length ORF7b in the surveyed samples using RNA-seq approach. The relative abundance of the splice junction sequences are shown the right panel of the figure. The numbers indicate the first and the last sites of splice junction sequences.

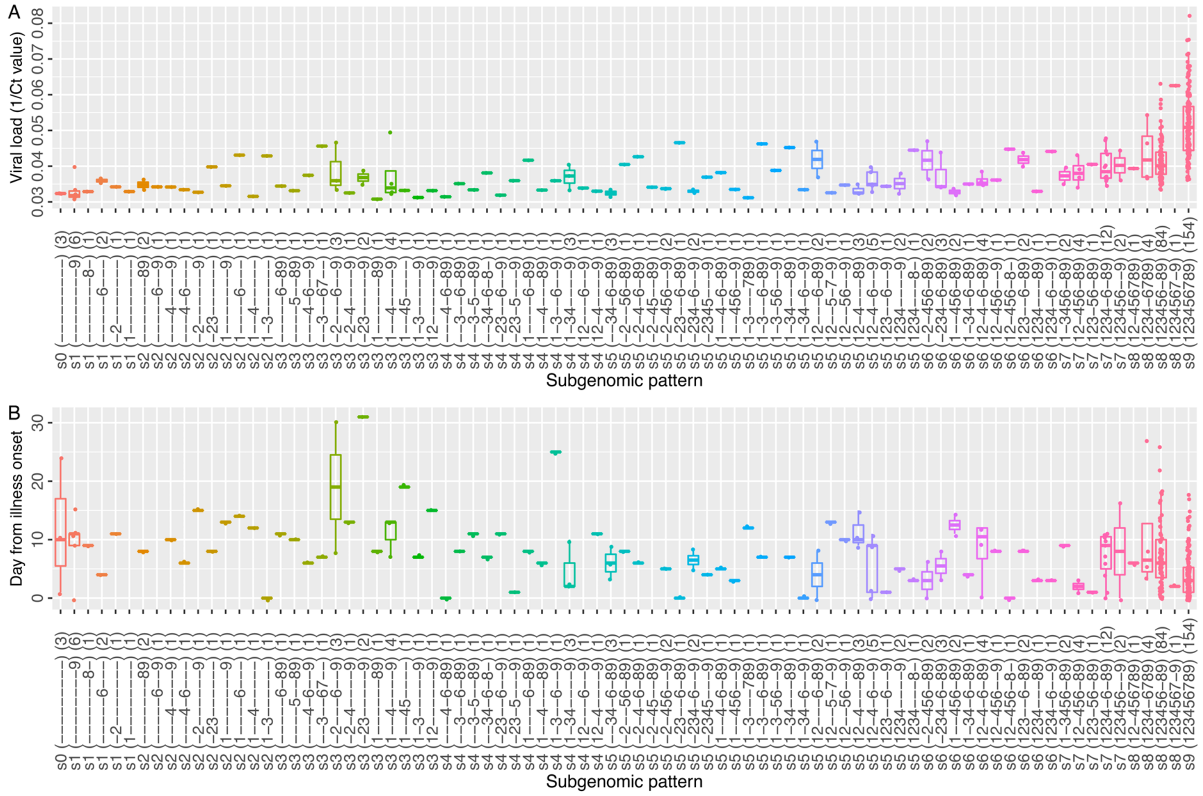

Figure S5. Subgenomic RNA profile in associate with viral load and time of specimen collection from illness onset. (A) Viral load indicated by genomic N Ct values. (B) Day of specimen collection from illness onset. Canonical ssubgenomic RNA pattern detected by the RNA-seq approach. “s0” – “s9” indicate the number of detectable canonical subgenomic RNAs. The numbers in the first bracket show the observation of individual subgenomic RNA (1, S; 2, ORF3a; 3, E; 4, M; 5, ORF6; 6, ORF7a; 7, ORF7b; 8, ORF8; 9, N). The number of detectable samples of each subgenomic RNA pattern was listed in the second bracket.

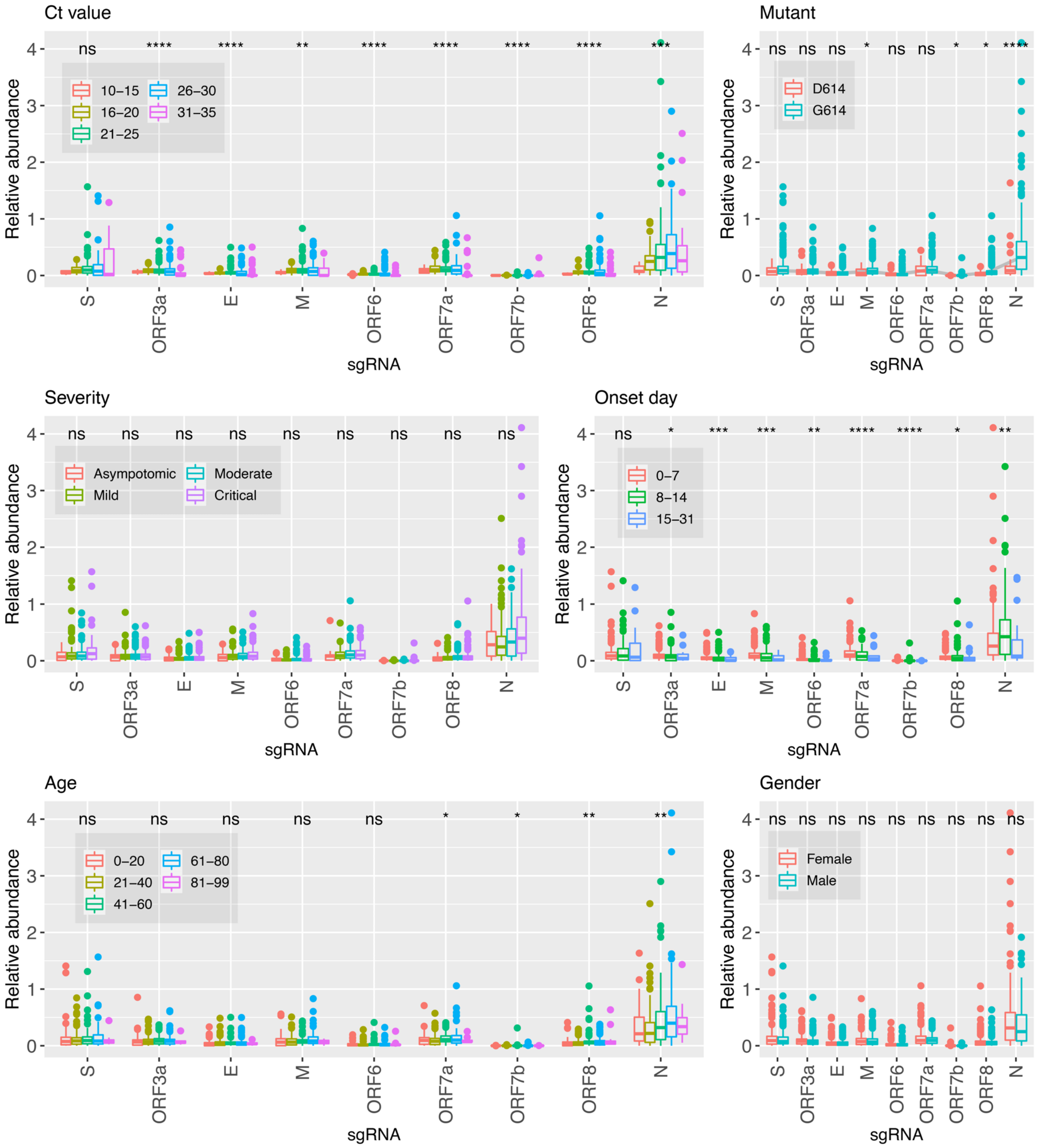

Figure S6. Comparison of relative abundance of canonical subgenomic RNAs inferred from RNA-seq data between samples with different clinical characteristics. Kruskal-Wallis Rank Sum test: * $p \leq 0.05$, ** $p \leq 0.01$, *** $p \leq 0.001$, **** $p \leq 0.0001$, ns no significance.

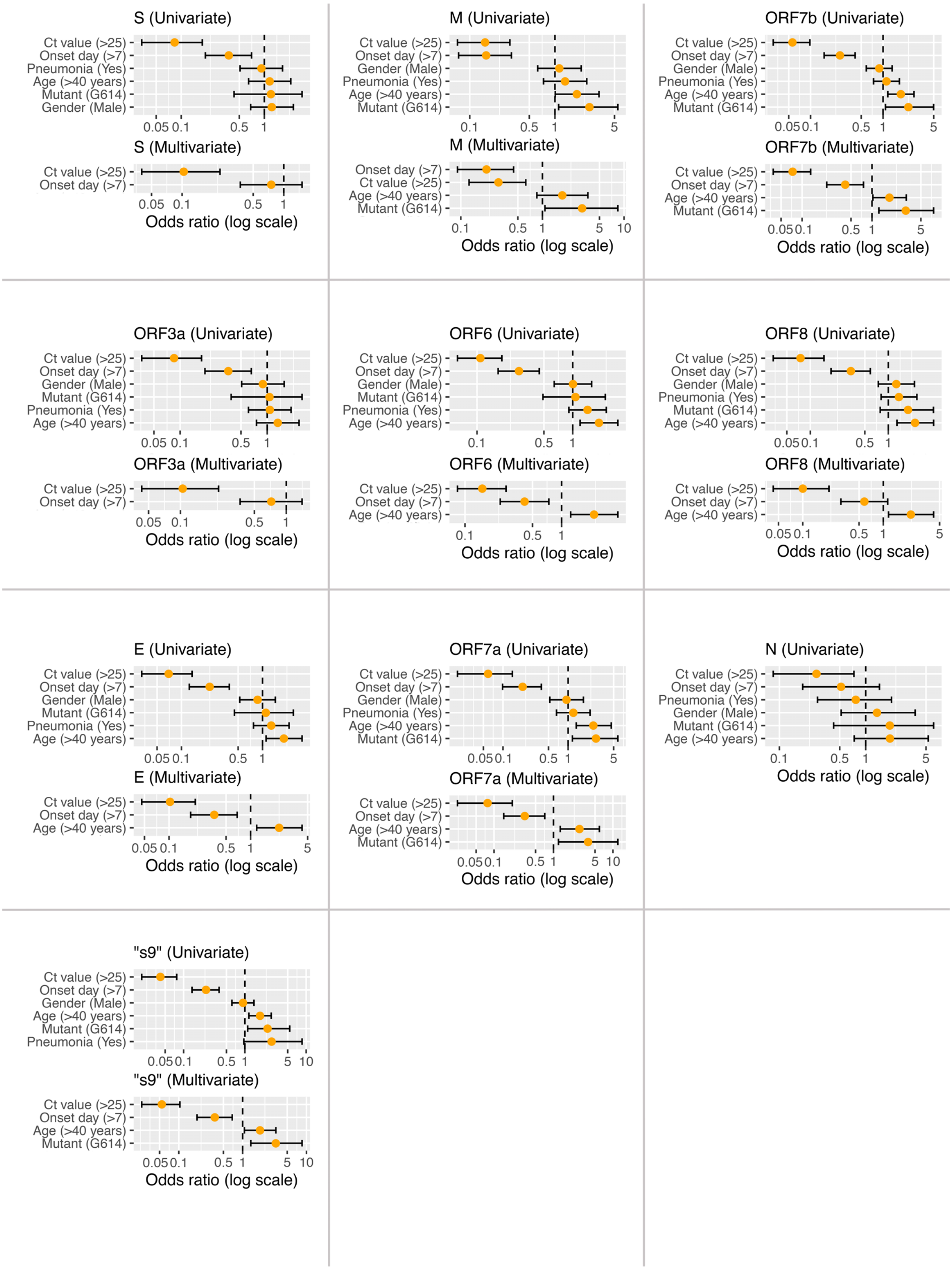

Figure S7. Univariate and multivariate regression analyses of association between subgenomic RNA patterns and clinical characteristics.

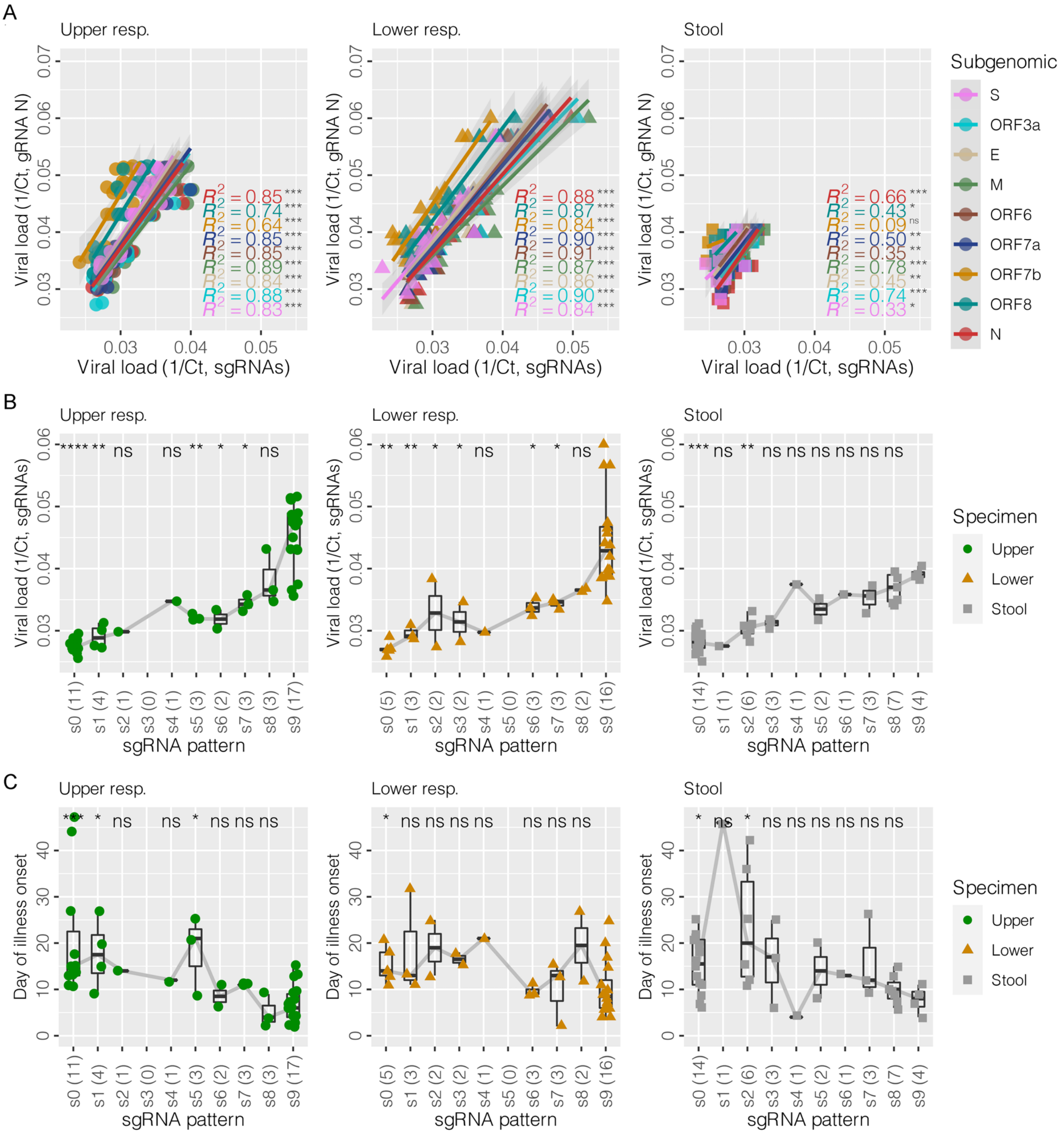

Figure S8. Profile of subgenomic RNAs revealed by RT-PCR in associate with viral load and time of specimen collection. **(A)** Scatter plot of correlation analysis showing positive association between genomic and subgenomic RNAs. **(B)** Detection of canonical subgenomic RNA patterns positively associated with viral load indicated by genomic RNA N Ct values. “s9” indicates a full spectrum of 9 canonical subgenomic RNAs, while “s0” means no detectable canonical subgenomic RNA. Mann-Whitney U test was performed between “s9” and partial subgenomic RNA pattern, with p values indicating * p $\leqslant$ 0.05, ** p $\leqslant$ 0.01, *** p $\leqslant$ 0.001, **** p $\leqslant$ 0.0001, ns no significance. **(C)** Association between canonical subgenomic RNA patterns and the day of specimen collection from illness onset.

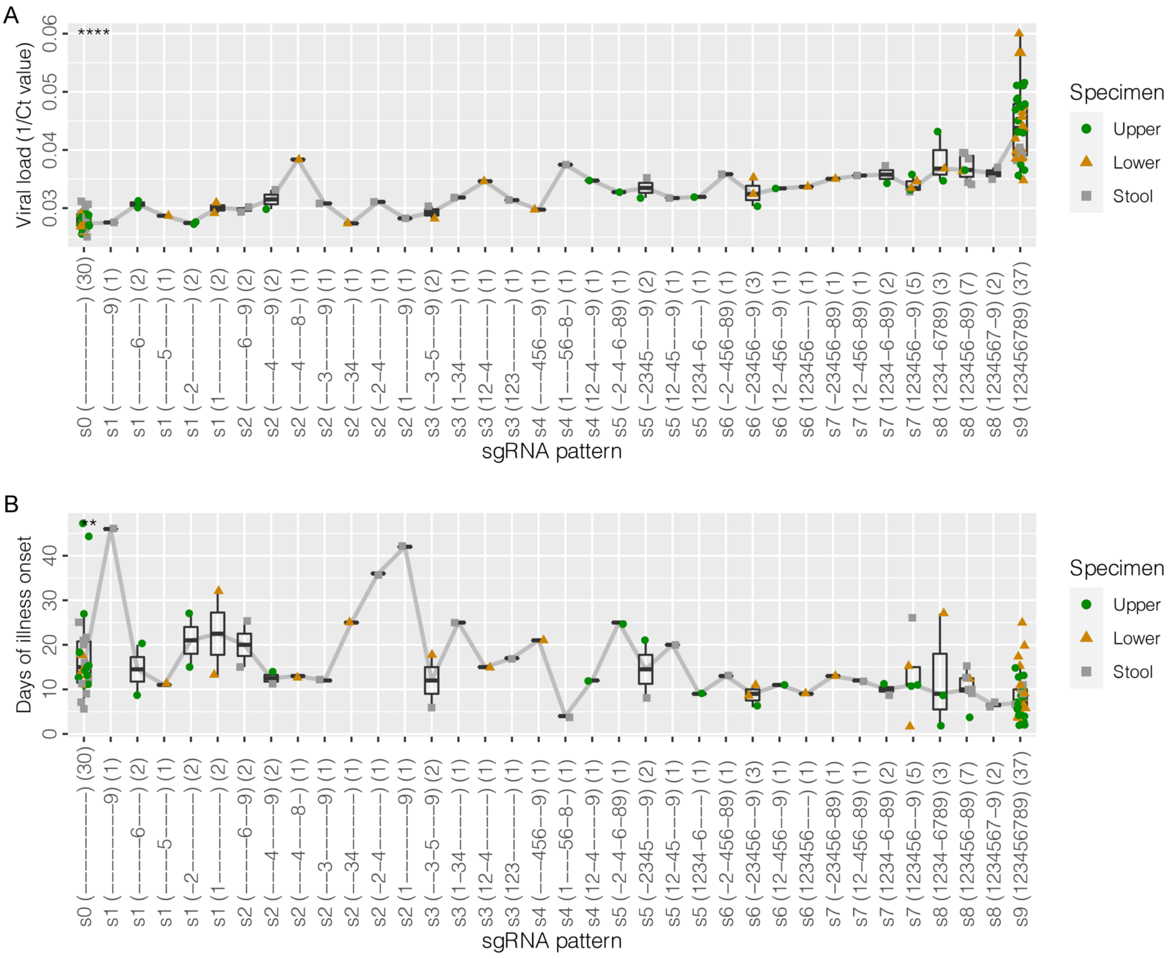

Figure S9. Profile of subgenomic RNAs revealed by RT-PCR in associate with viral load and time of specimen collection of individual sample. (A) Viral load indicated by genomic RNA N Ct values. (B) Time of specimen collection in days after illness onset. "s0" – "s9" indicate the number of detectable canonical subgenomic RNAs. The numbers in the first bracket show the observation of individual subgenomic RNA (1, S; 2, ORF3a; 3, E; 4, M; 5, ORF6; 6, ORF7a; 7, ORF7b; 8, ORF8; 9, N). The number of detectable samples of each subgenomic RNA pattern was listed in the second bracket.
